# Hospital and Institutional News

**Published:** 1919-05-10

**Authors:** 


### HOSPITAL AND INSTITUTIONAL NEWS.

### NEW MILITARY DIRECTORATES.

Sanction has just been given to a scheme put forward by the Director-General of the Army Medical Service, with a view to linking up the activities of all departments concerned with problems of preventive medicine, pathology, and tropical disease, which affect the health of our armies. The movement is in true harmony with the spirit of the times and we hope for its complete success, both in making full use of the wealth of new knowledge which the last few years have brought and in maintaining the standard of investigational and administrative work, which the R.A.M.C. with its temporary war complement has lately achieved. By such modernisation it is hoped to encourage not only officers who have lately specialised in these subjects to continue their activities therein, but also to encourage to enter the service younger medical men who have inclination towards these types of work. One notes that, following the war-time precedent, civilian experts are to be appointed on the Advisory Committees to each Directorate, representatives, in fact, of each civilian organisation working on the same lines. In the outline at present before us the scheme appears to hold the greatest promise.

### THE PARISH OF A POOR-LAW CHAPLAIN.

We are glad to see that the Newton Abbot Board of Guardians has rejected a proposal to appoint a chaplain without a salary, and has appointed the Rev. C. A. W. Russell, vicar-designate of Walborough, Devonshire, at a salary of £30 a year. In answer to objections, a speaker pointed out that the chaplain would have as much to do as the rector of a small parish. In fact, the inmates at present number 324, of whom 273 are described as belonging to the Church of England. For the forty-five Nonconformists a Free Church pastor has been appointed, with an honorarium of ten guineas a year. The chaplain's stipend is also an honorarium, except in name. He alone is technically "chaplain"; members of other bodies are technically "religious instructors." But we applaud the generosity of the Board and its prompter, a Church of England priest, in according to the unofficial chaplain a fee for his work.

### A MODEST SCHEME AT EXETER.

The extension of the Royal Devon and Exeter Hospital has become pressing in view of existing defects and the new work waiting to be done. First comes the need for a ward for disabled soldiers requiring prolonged treatment, to which the Ministry of Pensions has promised £2,000. But this grant, seconded by generous gifts, is still in itself only one-tenth of the total sum required. For, on the top of the above ward for soldiers, it is intended to build a children's ward containing fifty beds, with a flat roof for their open-air treatment. The waiting-list at present contains fifty children's names. The ear, nose, and throat cases also require departmental treatment. They need at least thirty beds, and though the soldiers' ward may eventually be used for them, in the meantime the inspection of schools in itself creates a steady flow of patients needing treatment. The nursing staff is housed at present in quarters admitted to be "as unsatisfactory as they can well be," and, with the new standard now set for their comfort, probationers will not be obtainable in any hospital that lags behind. For the latter purpose £2,500 is in hand; but Exeter and the district could so easily subscribe the remainder of the £20,000 asked for to complete the whole scheme that we should have hardly thought it necessary to offer to accept payments spread over three years. The waiting-list cannot be left to wait any longer. The time to start is now. With the signing of peace the whole sum should be raised in one day.

### MIRTH AND REMEMBRANCE.

In these days, when every one is apt to become a quick-change artist, when Prime Ministers become professional pianists at a moment's notice, it is refreshing to observe any one's return to normal life. The May number of the *Gazette* of the 3rd London General Hospital, Wandsworth, contains, for instance, a photograph of Derwent Wood, A.R.A., at work on a bust of M. Clemenceau. As we all know, during the war he made masks for facial injuries. That was a fact to be deplored as well as to be applauded, and we are glad to see him back at his original work. A sign of the times, too, is that Col. Bruce-Porter has handed over the command of the hospital to Major H. Fagan, the second in command. But though the late C.O. is demobilised and patients diminish and members of the staff disperse, the *Gazette* still continues, and the original contributors and draughtsmen contribute and draw as before. Memories begin to appear in its pages, and we believe that, when the last number shall appear, no one will regret it more than the contributors themselves. This is a joke they will appreciate—that the very disappearance of their magazine will be another cause for mirth. They will be remembered for it.

### UNREGISTERED DENTAL PRACTICE.

In the course of his speech at the National Dental Association dinner, Mr. J. A. Seddon, M.P., put forward the case of the unregistered dental practitioner. Referring to the activities of the Association he explained that its members belonged to a profession in which the Government had permitted a small section to be a privileged class. Under the approaching Ministry of Health Bill many new proposals would arise, and it behoved the Association to see that justice was impartially distributed. If only registered men were permitted to practise, there were tens of thousands of people who must go with defective teeth, because the number of men actually on the register was not nearly sufficient to meet the needs of the country. The whole matter is complex and difficult. With regard to the above statement the forecast is substantially true, and also we know that medically there is the most urgent need for the improved care of the nation's teeth. Yet, on the other hand, there are obvious objections to the extreme forms of quack dentistry. In the intended development of preventive rather than curative medicine there is no more important section than that affecting available dental care for the people. The Government will undoubtedly achieve an invaluable health service when a satisfactory solution is effected.

### "DUE PRAISE."

A CURIOUS letter has been published by the *Wigan Observer* from a medical man, Dr. Ferdinand Rees, in favour of State Hospitals. This cry for the "State" is so fashionable at the moment that it would hardly be worth notice were it not for certain sentences in the writer's letter which argue either very careless statement or considerable ignorance of facts. Replying to some other correspondent, Dr. Rees begins by supposing that the writer is "in the voluntary hospital business," a phrase hardly remarkable for tact or truth. The profession of medicine is not spoken of as "the medical business," nor are soldiers described as "the butcher's business," because there is a difference between a profession and a trade, especially between a man who has a merely commercial motive and he who has a super-selfish one. Dr. Rees then goes on to "give due praise" to the voluntary hospitals. "They have," he tells us, "done good work in the past in a small way"; in other words, he implies that "now" they do not do good work even in a small way. This is the "due praise" that Dr. Rees delights to give them. Even were there in the letter a considered argument for State Hospitals, and a real attempt to grapple with the defects of the voluntary system, such as the shortage of beds, we should have to condemn the short-sightedness of a man who forgets where he found his own training, and the ingratitude of an observer who slights the pioneer work of the voluntary system, because it has shown by its long record the extent yet to be covered and the national importance of what is being done. If Dr. Rees knows this, it is because the voluntary hospitals have taught him. If the nation now knows it, the nation is but echoing a cry that the voluntary hospitals were the first to raise. Nothing is more common than the failure to realise the value of existing institutions, nor vainer than the belief that if they are changed, the new will leave intact the virtues of the old. Before Dr. Rees decries "charity" and voluntary effort, he must prove that he understands what they mean.

### SPECIALIST JOURNAL FOR NURSES.

THE last number of the *Centralblatt für die gesamte Tuberkulose-Forschung* to reach this country contains mention of a special tuberculosis journal for nurses. The full title is "Fachzeitschrift für Krankenpflegerinnen und Fürsorgeschwestern, Lerausgegeben vom Fachverband der diplomierten Krankerpflegerinnen und Fürsorgeschwestern" (special journal for nurses and dispensary sisters, published by the Union of diplomaed nurses and dispensary sisters). It appears as a supplement to the Austrian tuberculosis dispensary journal, and deals on the one hand with the interests of those for whom it is written, and on the other with the scientific knowledge requisite in their work, giving mention of all new advances and facilitating what may be called post-graduate instruction. The two first numbers, already published, contain an article by Hofrat, Dr. E. Meder, the director of the nursing school in Vienna, on the history of the development of sick nursing in Austria. This publication is in all probability unique; at all events nothing analogous is to be found in any other language. It should be remembered, too, that in Germany and Austria the word "dispensary" does not connote solely tuberculosis, since similar institutions are in operation dealing with venereal disease and with alcoholism. The "V.D. clinic" is a term beginning to be heard here, but clinics for drunkards have not yet been proposed, although in the North of England, and especially in Scotland, there would be great room for them.

### WHO SHOULD RUN A BIG HOSPITAL?

A CHARMING speech was made by Capt. A. W. Clarke, C.B.E., at the annual court of the Dreadnought Hospital, in which his praise of Sir Perceval Nairne and of Mr. Michilli was so salted with chaff as to keep its savour and their modesty unadulterated. After mentioning Sir Perceval's services to the sick and wounded merchant seamen, and his unruffled good temper in committees, Capt. Clarke addressed himself to Mr. Michilli, and incidentally had something to say about the influence of the secretary in hospital life. "If you think," he said, "that the chairman, the vice-chairman, and the committee do what they like, you are mistaken. We all do what the secretary tells us. When you have a secretary like Mr. Michilli, you are wise to obey him, for he is a prince in his profession." This tribute is well deserved, for the men of whom it can be said as truly are still few. It is odd that the profession of hospital administration has lagged behind other branches of hospital life in securing the due rate of payment, and the best educated men. The truth is that hospitals have developed more quickly than the type of man who administers them. A modern hospital has so many departments, its medical staff is so much increased, that the larger hospitals here, as in the United States, are beginning to favour medical superintendents. This does not relegate the secretary to a second place. It merely recognises a division of labour. Finance and business can be left to the laymen. But when, in addition, a hospital becomes a great medical centre calling for medical direction of a high type, the appointment of a medical superintendent is a need that no big hospital can fail to admit. It is one of the lessons of the war.

### AN ECHO FROM 1913.

THE people of Lowestoft have decided on a hospital as a war memorial. In the absence of the hospital's president, Lord Stradbroke, who is in

Egypt, Colonel A. G. Lucas, in addressing a public meeting, reminded his audience that in 1913, "a gentleman went round the country to report on the hospitals and condemned Lowestoft hospital from top to bottom." In consequence, the then committee decided to improve the hospital, but the work had been suspended because of the war. A scheme had been prepared by an architect, which was expected to cost £30,000. He, Colonel Lucas, had been assured by Sir George Newman that there was nothing in the scare of nationalisation, as, we may remark, The Hospital stated as soon as it arose. The Government, Sir George had told him, would be willing in every way to support the voluntary system. Sir George, in a later letter, assured him further that Dr. Addison had stated that there was nothing to fear from the Ministry of Health in regard to building hospitals as war memorials. The new hospital would provide wards for private patients, and it would be far more economical to build a new institution than to try to patch the old one. The fruits of the report of 1913 have been delayed, but the value of the work then done is fully vindicated.

### THE BERKSHIRE WAR MEMORIAL

Mr. Charles Steward Smith has drawn an interesting plan for the proposed Nurses' Home in connection with the Royal Berkshire Hospital, which may yet be adopted as the war memorial of the county. The site in Craven Road adjoins the hospital, which, were it added, would become virtually a square island, surrounded by thoroughfares, with only a Presbyterian church to occupy one corner of the front.

### HOME FOR HOSPITAL SERVANTS.

There are several interesting points in the proposed reconstruction of Norfolk and Norwich Hospital. Among them is a suggested home for the domestic staff, at present housed in part of the museum and in the old isolation wards—an emergency measure condemned even by those who sanctioned it. A new building for the domestic staff is proposed, and an appeal is being issued. The home for the domestic staff will be a three-storey building at the far end of the garden behind the Nurses' Home. It will consist of 24 rooms, one for each maid, a sick-room on each floor, bathrooms and lavatories. The space gained by the removal of the maids will be hardly sufficient to extend the overcrowded departments. We are glad to see that the hospital standard is at last being applied to the domestic staff. To improve nurses' quarters was the work of years and required persistent advocacy. The effect of its success has been to raise the hospital standard all round.

### A ROMANCE OF SWANSEA HOSPITAL.

In the History of Swansea Hospital, by Mr. David Salmon, issued in connection with the centenary of the hospital, there is a curious story of a legacy which was never paid in full but which established the hospital on a sound basis. The generous intentions of the testator were undoubted. He was Dr. Edwards, the first resident surgeon, who in 1831 left the Infirmary (as it was called in those days) his library, manuscripts, and instruments, and the sum of fifteen hundred pounds payable after the death of his wife. The first announcement of the will gave a scheme for expansion such an impetus that it proceeded and succeeded. It was not until success was assured that it was found that Dr. Edwards had, for some unknown reason, joined with two other men in a bond of £4,482. The first man had died leaving nothing; the second, though alive, owned nothing, so there was a probability that most of the money would have to come out of the doctor's estate. How much did eventually come out of it the records do not show, but after nine years of correspondence and worry the hospital eventually received £335. The library was handed over intact, but cost £122 to house until the hospital could find room for it.

### NEW HOSPITAL FOR RHONDDA DISTRICT.

Notwithstanding the increase of beds from 24 to 43, the building of a new theatre and the approaching installation of an *x*-ray apparatus, the Porth Cottage Hospital is felt to be insufficient. The governors have decided to found a new hospital of 200 beds or so for the district.

### AUXILIARY HOSPITALS' SURPLUS STOCKS.

Mr. W. G. Gibbs, hon. secretary of the Leicester and County War Hospitals Committee, has reported a ruling of the Charity Commissioners in regard to the disposal of the surplus stocks of auxiliary hospitals. With respect to gifts in kind still in these institutions, "the Commissioners approve of the proposal that the surplus books and other goods should be applied primarily to hospitals and similar institutions where sailors and soldiers are being treated." Other institutions are allowed to benefit only when "the primary application becomes impossible." The general regulation is that surplus funds can be disposed of only when the war charity is "wound up" on completing its work, when the wishes of the subscribers and of the Commissioners have to be consulted.

### A NEW BARONET'S HOSPITAL INTERESTS.

Mr. James Marr, C.B.E., who was among the recipients of a baronetcy in the list of honours published last week, was chairman of Monkwearmouth and Southwick Hospital, Sunderland, from 1908 to 1914. During his chairmanship many important improvements were carried out, and his influence was instrumental in attracting to the hospital substantial financial support. Sir James Marr continues to take a live and sympathetic interest in all that concerns the institution.

### SERVICES TO GREAT ORMOND STREET

Dr. George R. Pirie, of Calgary, Alberta, Canada, who throughout the period of the war has acted in the triple capacity of resident medical superintendent, medical registrar, and casualty medical officer to the Hospital for Sick Children, Great Ormond Street, was presented with tokens of regard before leaving London on Saturday to sail for his home. The presentations from the committee of management and the medical committee took the form of a silver casket and a dressing case respectively, with a suitable inscription on each. High testimony was given to the valuable services Dr. Pirie rendered to the hospital in the trying times of the war, and in assisting to maintain the high reputation for efficiency which it has been the hospital's proud distinction to hold. Dr. Pirie, in acknowledging the gifts, spoke of the pleasure the work at Great Ormond Street had given him. He had become deeply attached to the hospital.

### STAFF CHANGES AT CHESTERFIELD.

The resignation of Dr. Albert Green from the position of honorary surgeon to the Chesterfield Royal Hospital, and the granting of his request to be placed upon the consulting staff, has led to several important decisions. Having resigned because of the growth of his private practice and of the "increasing demands of the hospital," Dr. Green's action led the board to revise the appointments. It has, therefore, decided to appoint three new honorary surgeons, a pathologist, and an additional honorary dental surgeon.

### A RETIRING CHAIRMAN'S CRITICISMS.

On retiring after eight years from the chairmanship of the Royal Portsmouth Hospital, Mr. T. H. F. Lapthorn, who has spent much time, apart from his official duties, at the hospital, made a valedictory criticism which is well worth attention. Having frequently referred to the unsatisfactory position in which the hospital has found itself, and to the lack of support of which it has complained, we note Mr. Lapthorn's opinions with interest. He recalled the establishment of the governors about 1902; they were part of the reorganisation and consisted of old friends for whom no place could be found on the board of management. The governor's chief responsibility was, he said, to raise the necessary funds, so that the executive work could be left to the board of management. Mr. Lapthorn did not think that the governors, as a body, had risen to their responsibility. This had been left to the committee of management and a few others. If further reorganisation took place, he hoped this fact would be remembered. He further admitted his detestation of the ticket system, but defended it as a temporary financial measure, and, as a farewell piece of advice, recommended the amalgamation of the hospital with the Eye and Ear Hospital. We wish all chairmen made equally good use of their last official opportunity.

### A LATE MEDICAL SUPERINTENDENT'S FORTUNE.

Dr. John Edward Montague Finch, M.P., of Stoneygate, Leicestershire, from 1869 to 1911 resident medical superintendent of Leicester Borough Asylum, who died in February, aged seventy-seven years, left estate valued at £72,056. He bequeathed £5,000 to "the Mayor, Aldermen, and Burgesses of Leicester for the endowment of a university for Leicester in remembrance of my long services as Medical Superintendent of the Borough Asylum." To the Leicester Royal Infirmary he left £250.

### THE PETERBOROUGH REVIVAL.

We are glad to see that the people of Peterborough have awakened from their dream, and that the scheme to raise £70,000 for a new infirmary as a War memorial is to go through. The example of the Mayor, Capt. C. T. Veigette, and of Dr. Collins has proved infectious, as such vigorous leading always does. The working-men are prepared to do their share in maintaining the new hospital, and we hope that the larger rather than the smaller sum will be asked for, now that the importance of securing it is realised at last.

### POULTRY AND GERMAN PRISONERS.

Should the master of a workhouse be allowed to keep poultry for himself in the institution's grounds? The Newton Board of Guardians does not object, but an old member of the Board feels so strongly on the point that he has resigned, and in doing so has sent the following letter: "As the Board has consented to allow the master to keep his poultry-farm for his own private use and pleasure on the workhouse premises (after the Agricultural Committee had refused him such permission), and using the inmates' labour to attend to them, whilst at the same time employing German prisoners of war to till the workhouse garden, I feel compelled to show my disapproval by resigning." Unless the Board has to square accounts with the Agricultural Committee, its decision is presumably sufficient. But this is not the first time we have heard complaints that private persons were using the labour of German prisoners for private profit.

### THIS WEEK'S DRUG MARKET.

While an advance in the duty on alcohol was expected, the advance that has actually been made is much higher than had been generally anticipated. Presumably a rebate equivalent to the advance will be allowed on spirit used in the manufacture of scheduled medicinal preparations, but a number of articles will not be subject to the rebate, and prices for such are bound to be higher. The Budget statement having removed one of the uncertainties, the drug market is slightly more active; but until peace is actually signed it is doubtful if business will begin to assume a more normal shape. Prices, generally speaking, will most probably continue to recede. In many cases values are still too high to tempt purchasers who are not in an immediate hurry for supplies. Turkey opium is now arriving, and we may expect to see a fall in prices not only of the raw material but also of its derivatives. Values of menthol, peppermint oil, and camphor are firmly maintained. Linseed oil is dearer. Citric acid is in better request and has a slightly upward tendency in price. Included in the list of articles with a downward tendency are acetic acid, tartaric acid, almond oil, enonymin, thymol, aloin, podophyllin, atropine sulphate, lemon oil, salicylates, aspirin, resorcin, paraldehyde, and salol.

